# Editorial: COVID-19 and entrepreneurial mindset

**DOI:** 10.3389/fpsyg.2022.1084783

**Published:** 2023-01-12

**Authors:** Eijaz Ahmed Khan, Mohammad Shamsuddoha, Shahriar Sajib, Md. Nuruzzaman, A. K. M. Ahasanul Haque

**Affiliations:** ^1^College of Business, Law & Governance, James Cook University, Townsville, QLD, Australia; ^2^School of Management and Marketing, Western Illinois University, Macomb, IL, United States; ^3^UTS Business School, University of Technology Sydney, Sydney, NSW, Australia; ^4^Department of Marketing, Rajshahi University, Rajshahi, Bangladesh; ^5^Department of Business Administration, International Islamic University Malaysia, Kuala Lumpur, Malaysia

**Keywords:** entrepreneurial mindset, entrepreneurial personality, cognitive adaptability, entrepreneurial cognition, COVID-19

The entrepreneur's importance is well-recognized in the economy, especially during critical times. The COVID-19 pandemic is already considered to be one of the biggest socio-economic crises in history. As it is ongoing and bringing many business-related uncertainties, it is very challenging to foresee its impact on the future. However, despite the challenges created, the COVID-19 pandemic also created entrepreneurial opportunities (Ketchen and Craighead, 2020; Emami et al., 2022).

Understanding this phenomenon has become an important issue in entrepreneurship research. Entrepreneurs are highly interested to explore the major challenges arising during the COVID-19 pandemic and to develop appropriate strategies to exploit opportunities. However, to forecast and determine the best course of action on how to respond to challenges related to the COVID-19 pandemic, entrepreneurial energy and mindset is required (Engel et al., 2021; Segares et al., 2022). Therefore, the purpose of this Research Topic is to comprehensively examine and describe issues linked to “*COVID-19 and entrepreneurial mindset*.”

To date, a large number of scholars have addressed different aspects of the entrepreneurial mindset, contributing to the understanding of its qualities and attributes (Kuratko et al., 2021). Scholars have also provided insights into its association with coping mechanisms for uncertain and complex environments (Pidduck et al., 2021). Nevertheless, scholarly studies were not yet outfitted to offer comprehensive knowledge on how to deal with such a context as the COVID-19 pandemic (Daspit et al., 2021). The COVID-19 pandemic, along with the new nature of business environment, complexity, and uncertainty, caused a shift in work requirements. If entrepreneurial mindsets can be discovered and stimulated, it could inspire businesses' existence and progress (Pfeifer et al., 2016).

In the current Research Topic, we covered a wide range of topics in the four manuscripts inlcuded. The first was an article from Sardar et al. examining the impacts of the COVID-19 pandemic on restaurant owners and their businesses. The study interviewed a total of 22 restaurant owners and found that due to the necessary measures taken to protect against the pandemic, customers were psychologically affected and felt uneasy to use the services in loco. The study's findings could help decision makers related to the restaurant business to develop and implement concurrent business strategies and policies. The study also explored dramatic shifts, such as the increase in online delivery modes and the physical distancing taking place in business operations, which put significant pressure on restaurant owners.

The next article, from Hassan et al., identified the role and importance of entrepreneurial networks among tertiary-level students, as well as entrepreneurial education, in building social entrepreneurial intention. A total of 392 students attending public and private institutions in Bangladesh were selected using a convenience selection technique and then asked to complete a self-administered questionnaire. During data analysis, the study used structural equation modeling with partial least squares (PLS-SEM). Results showed that entrepreneurship education positively influences students' social entrepreneurial intention. Besides, entrepreneurship education positively impacts entrepreneurial social networks. Finally, entrepreneurial social networks have a positive relationship with students' social entrepreneurial intention. Overall, the study confirmed the partial mediation of entrepreneurial social networks. This study added valuable understanding to the role of social networking in entrepreneurial educational organizations, which is beneficial for creating practical education strategies. The article by Rehman et al. analyzed entrepreneurs' dispositional factors (start-up behavior) as an underlying mechanism to bridge trust and entrepreneurial success. The study applied time-lagged data collection from 505 owners and top executives of the manufacturing industry in Pakistan. The study suggested that start-up behavior is the underlying mechanism through which entrepreneurs' trust influenced entrepreneurial success. The study also analyzed instances when trust matters more in predicting entrepreneurial success at low or high entrepreneurial strategy. This study contributed to the literature on entrepreneurial strategy for its conditional indirect moderated impact on start-up behavior and moderated mediation impact on firm entrepreneurial success. This study also concluded that the start-up behavior of manufacturing industry business owners was an underlying mechanism through which trust influenced their entrepreneurial success. The last study, by Susanty et al., outlined a model to examine SMEs' poor performance due to the impact of the COVID-19 pandemic. Short telephone interviews and web-based closed questionnaires were applied to collect data. The study identified and explained the nature of several business operation indicators arising from the COVID-19 pandemic that could help decision makers when considering business operation interventions.

The need for entrepreneurial mindsets is a critical component of social and economic development. Very few researchers have focused on building robust studies on entrepreneurial mindset. This editorial highlights the necessity of understanding entrepreneurs' mindsets in this era of the global health pandemic caused by COVID-19. An entrepreneurial attitude can be useful in a range of situations, from non-profit and social endeavors to workforce development and academic achievement (Canziani and Welsh, 2021). It is not simply about establishing a business. It deals with the capacity to identify chances for problem-solving, without the need for unique skills, extensive access to venture funding, or the development of ground-breaking technologies (Fernandez, 2021). An entrepreneurial mentality begins when an individual connects his or her interests and skills with the primary purpose of bettering themselves or their immediate surroundings, which in turn motivates such behavior.

## Author contributions

EK supervised the whole process, while MS and SS managed the reviewing process. All authors contributed to the article and approved the submitted version.
